# Physical Activity, Telomere Length, and Cardiometabolic Syndrome in Older Women: The Cardiovascular Health Study

**DOI:** 10.1177/01939459251387798

**Published:** 2025-11-29

**Authors:** Jeni Page, Melissa Richard, Elizabeth Lyons, Elizabeth Baumler, M. Terese Verklan, Michelle C. Odden, Elizabeth Lorenzo

**Affiliations:** 1 School of Nursing, University of Texas Medical Branch, Galveston, TX, USA; 2 School of Nursing, Texas Tech University Health Sciences Center, Lubbock, TX, USA; 3 Baylor College of Medicine, Houston, TX, USA; 4 School of Health Professions, University of Texas Medical Branch, Galveston, TX, USA; 5 McGovern Medical School, University of Texas Health Science Center at Houston, Houston, TX, USA; 6 Department of Epidemiology and Population Health, Stanford University, Stanford, CA, USA

**Keywords:** physical activity, telomere length, cardiometabolic syndrome, aging

## Abstract

**Background:**

Cardiometabolic syndrome (CMS) affects approximately 39% of US women and increases risk for cardiovascular disease, diabetes, and stroke. Telomere length, a biomarker of cellular aging, is associated with CMS and may be influenced by physical activity (PA), potentially mitigating telomere shortening. Black women have higher CMS prevalence compared to white women and may exhibit distinct telomere length dynamics influenced by interactions between CMS and PA.

**Methods:**

Data were drawn from the Cardiovascular Health Study and included black and white women who provided data on PA, CMS risk factors, and telomere length (N = 417). Linear regression models examined the associations between CMS, PA, and telomere length cross-sectionally and prospectively, with analyses conducted separately for black and white women.

**Results:**

No associations between PA and telomere length were observed in black or white women. High-density lipoprotein levels <50 mg/dL were significantly associated with longer telomere length among black women, but no other CMS risk factors were associated with telomere length. There were no interaction effects between PA and telomere length when accounting CMS risk factors.

**Conclusions:**

PA did not significantly relate to telomere length in older black or white women, and CMS risk factors did not influence the PA–telomere length association. The association between low high-density lipoprotein levels and longer telomere length in black women warrants further investigation. Future research should consider larger samples, objective PA measures, and additional confounders to clarify the roles of PA and CMS in telomere length dynamics among older black and white women.

## Introduction

Cardiometabolic syndrome (CMS) is a group of metabolic risk factors that affects 1 in 3 adults in the United States, leading to substantially increased risk of cardiovascular disease, diabetes, and stroke.^1^ CMS is defined using criteria from the modified National Cholesterol Education Program Adult Treatment Panel III (NCEP ATP III),^2^ which includes the presence of 3 or more of the following conditions: (1) abdominal obesity measured by high waist circumference, (2) high blood pressure, (3) high fasting blood glucose, (4) high triglycerides, and (5) low high-density lipoproteins.^3^ Prevalence of CMS has risen over the past several years, with approximately 39% of women diagnosed in the United States,^4^ with incidence only increasing with age.^5^ Moreover, black women are even more impacted with black women exhibiting higher rates of CMS compared to their white counterparts at all ages.^6^

Telomeres are repetitive DNA–protein complexes that cap the ends of linear chromosomes, serving both to protect and maintain genomic stability.^7^ Telomere length progressively shortens with aging due to its known role in cellular senescence,^8^ making it an important biomarker of cellular aging as well as an indicator of health and the lifespan of an individual.^9^ Research indicates a potential correlation between telomere length and various risk factors associated with chronic disease.^10^ The association between telomere length and chronic disease is particularly relevant in the context of CMS. Accelerated telomere shortening is believed to be a result of oxidative stress and chronic inflammation,^11^ mechanisms strongly linked to CMS development.^12^

Previous research has supported the relationship between CMS risk factors and telomere length, underscoring that manifestations of CMS risk factors align with abnormal telomere shortening.^13-15^ Interestingly, physical activity (PA) appears to exert an inverse effect on telomere shortening.^16^ Numerous studies have identified longer telomere length in individuals engaging in higher levels of PA, presumably due to a reduction in oxidative stress and chronic inflammation.^17-19^ The positive impact of PA on telomere length is crucial in the context of chronic disease, including CMS. Additionally, research has shown variations in telomere length based on factors such as sex, race/ethnicity, and age. However, data on race and ethnic variations are conflicting. While some studies have shown that black women possess longer telomere length compared to white women,^20,21^ others have suggested that with age, black women experience more pronounced telomere attrition.^22,23^ Accelerated telomere attrition is thought to arise from the heightened social stress experienced more commonly by minority populations.^24^ Over time, the compounded stress can lead to increased oxidative stress and chronic inflammation, culminating in a more rapid reduction of telomere length.^25^

Sex-specific biological, hormonal, and sociocultural differences strongly justify a focused analysis of women. Women experience steeper age-related increases in CMS prevalence than men, with greater prognostic significance for cardiovascular disease and mortality.^26^ CMS also presents differently by sex: women are more likely to meet diagnostic criteria through increased waist circumference and low high-density lipoprotein (HDL),^27,28^ both of which have sex-specific thresholds.^29^ Hormonal changes, particularly the decline in estrogen during menopause, contribute to increased visceral fat, insulin resistance, and inflammation, key drivers of CMS.^26,30,31^ Estrogen also plays a protective role in telomere biology by upregulating telomerase activity and reducing oxidative stress,^32-34^ suggesting that menopause may simultaneously elevate CMS risk and accelerate telomere attrition.^35^

These mechanisms are further influenced by race-specific disparities. Black women exhibit disproportionately higher rates of CMS, obesity, hypertension, and diabetes compared to their white counterparts,^36,37^ and also report lower levels of PA, particularly in later life.^38^ These factors are compounded by unique sociocultural exposures, including chronic stressors such as racism and discrimination, which may affect biological aging through inflammatory and neuroendocrine pathways.^22,39^ Despite these disparities, black women remain underrepresented in biomarker research,^40,41^ and most studies of telomere length and CMS either include primarily male participants, rely on predominantly white cohorts, or fail to stratify by sex.^42,43^ These gaps underscore the need for research focused explicitly on women, particularly racially diverse women, to elucidate the distinct biological, behavioral, and social contributors to CMS risk and cellular aging.

While studies have independently linked CMS risk factors and PA to telomere length, few have examined their combined influence in women. Prior research on racial differences in telomere length has yielded inconsistent findings, and black women remain underrepresented in aging biomarker research. Moreover, most existing studies do not integrate behavioral and biological risk factors in relation to cellular aging. This study was designed to examine these interrelated constructs in a racially diverse cohort of women.

### Problem and Purpose

This secondary data analysis examines the association of PA and CMS with telomere length in black and white women. Guided by Kang’s Expanded Biobehavioral Model,^44^ the study conceptualized PA as a behavioral factor, CMS risk factors as biological indicators, and telomere length as a cellular-level measure of individual health status. The model helped inform how PA and CMS risk factors may influence telomere length, guiding the selection of variables and the development of study hypotheses. It was hypothesized that (1) Telomere length among black and white women would be associated with differences in the presence of CMS or CMS risk factor presence and (2) PA participation would be associated with telomere length differences in black and white women.

## Methods

### Data Source

The Cardiovascular Health Study (CHS) is a longitudinal, observational study funded by the National Heart, Lung, and Blood Institute with the main objective of discerning risk factors associated with coronary artery disease and stroke in older adults.^45^ Data were collected from 4 counties across the United States: Sacramento county, California; Washington county, Maryland; Forsyth county, North Carolina; and Pittsburgh, Pennsylvania.^46^ All the participants provided informed consent at baseline and prior to data collection at interval times throughout the study. Institutional review board approval was obtained from all institutions involved in the study.

### Population

The initial cohort from the CHS was enrolled between 1989 and 1990, comprising 5201 participants.^46^ To increase participation of black individuals in the study, an additional cohort of predominantly black participants was added from 1992 to 1993, totaling 687 participants. Random sampling of Medicare eligibility lists from each of the four Field Centers was used to select participants. Participants had to meet the minimum 65 years age requirement, not be wheelchair-bound or undergoing chemotherapy or radiation therapy, not be on hospice, and could not be institutionalized. For all the participants, informed consent was obtained and then each participant underwent a comprehensive baseline assessment including obtaining anthropometric and physiologic measures, blood samples, and providing questionnaire responses.

### Participant Inclusion and Exclusion Criteria

Participants included for analysis were drawn from an existing CHS ancillary study of telomere length. A total of 1675 participants were randomly selected from among participants who provided consent for DNA preparation/use and had a suitable DNA specimen available from the 1992 to 1993 study visit.^10^ Follow-up DNA specimens at the 1997 to 1998 CHS visit were selected for the 1675 participants who survived until 1997 to 1998 and had adequate DNA available at the follow-up visit (n = 702). The current analysis was restricted to black and white women who had completed the PA questionnaire and had CMS risk factors measured at both the 1992 to 1993 visit and the follow-up visit 4 years later in 1996 to 1997 (n = 417). The 1992 to 1993 study visit serves as the baseline for this analysis and will be referred to as the baseline visit hereafter.

### Measures

#### Cardiometabolic Syndrome Risk Factors

CMS and CMS risk factors were defined based on the criteria from the modified National Cholesterol Education Program Adult Treatment Panel III,^2^ which is one of the most widely used definitions for CMS in US-based populations.^2^ These criteria were selected for their relevance for assessing metabolic risk in US-based cohorts and their consistency with public health guidelines.^47^ Clinical and laboratory measurements were collected during morning clinic visits following a required 12-hour fast. Venipuncture was performed early in the examination. Serum samples were used to analyze fasting glucose and insulin using the Kodak Ektachem 700 Analyzer (Eastman Kodak Company, Rochester, NY, USA). Plasma lipid measurements, including HDL and triglycerides, were conducted using the Olympus Demand system and standardized to CDC protocols. Samples were aliquoted, frozen at −70°C, and shipped weekly on dry ice to the Central Blood Analysis Laboratory.^27^ Quality control procedures and centralized monitoring have been previously described.^45^

Each component was treated as a separate risk factor and was dichotomized as absent (0) or present (1) using the NCEP ATP III-specific abnormal cut-off points: high waist circumference >35 inches; high blood pressure which included systolic blood pressure ≥130 mmHg and/or diastolic blood pressure ≥85 mmHg combined into a dichotomous variable; high fasting blood glucose ≥100 mg/dL; high triglycerides ≥150 mg/dL; and low high-density lipoprotein <50 mg/dL. In data analyses, participants were coded as positive for a CMS risk factor if they met the abnormal clinical cut-off or were taking medication to treat that condition, including antihypertensives, antihyperglycemic medications, and lipid-lowering agents. CMS was operationalized as a dichotomous variable, defined as absent (0) if <3 risk factors or present (1) for ≥3 risk factors. CMS risk factor variables were collected at baseline (waist circumference, systolic blood pressure, diastolic blood pressure, fasting blood glucose, triglycerides, and high-density lipoprotein), and 4 years later (waist circumference, systolic blood pressure, diastolic blood pressure, and fasting blood glucose); lipid data were not collected at the 4-year follow-up, which is acknowledged as a study limitation.

#### Physical Activity

The CHS used a modified version of the Minnesota Leisure-Time Activity Questionnaire to assess PA (content validity for women: 0.36; test–retest reliability: 0.79-0.88).^48-50^ The questionnaire collected PA data at baseline and queried about the frequency of sessions in a 2-week period of time and duration in minutes per session of 15 different activities. Intensity was determined using the 2011 Compendium of Physical Activities by calculating the metabolic equivalent and then categorizing the PA type as light-, moderate-, and vigorous-intensity.^51^ Activities were then classified into moderate-intensity PA and vigorous-intensity PA consistent with prior research.^52^ The frequency and duration of sessions were then used to compute the weekly minutes of moderate-intensity PA and vigorous-intensity PA. Adherence to the PA national guideline recommendations was then measured using 2 dichotomous indicators. Moderate-intensity PA was coded as a dichotomous variable (<150 minutes of moderate-intensity PA per week/≥150 minutes moderate-intensity PA per week) and vigorous-intensity PA was coded as a dichotomous variable (<75 minutes of vigorous-intensity PA per week/≥75 minutes vigorous-intensity PA per week), with each statistical model including both PA adherence indicators.

#### Telomere Length

Telomere length was quantified in kilo base pairs (kbp) and was measured as the mean length of the terminal restriction fragment from DNA extracted from peripheral leukocytes, as detailed by Fitpatrick et al.^11^ Participants who had consented to DNA analyses and had at least 12 μg of DNA available were included in the study. The integrity of DNA was evaluated through electrophoresis and stained with ethidium bromide. The mean terminal restriction fragment length was determined using the Southern blot method, which has been previously described.^53,54^ Each sample underwent telomere length measurement twice, and the mean of the two measurements was used for statistical analyses, achieving a Pearson correlation coefficient of 0.97 for the replicates, and a mean coefficient of variation at 1.5% for paired sets.^10^ Throughout the process, the laboratory personnel performing the telomere length measurements were blinded to participant characteristics.

#### Confounders

In preliminary analysis, a range of potential confounders known to be associated with telomere length based on existing literature were considered. These confounders included age, body mass index, highest level of education (<high school, high school graduate, some college, >college), marital status (single, married, separated/divorced, widowed), and annual income (<$5000-$24 999, $25 000-$49 999, ≥$50 000). Additionally, cigarette smoking status (never, former, current) and alcohol use (none, <1 alcoholic drink per day, ≥1 alcoholic drink per day) were considered. Age was found to be the only confounder that was found to be significant between PA and telomere length, and CMS or CMS risk factors and telomere length, and was therefore retained as a confounder in all linear regression models.

### Statistical Analysis

Chi-square analyses and independent sample *t*-tests were used to examine bivariate associations. These tests assessed the relationships between the sociodemographic variables, PA, CMS, and/or CMS risk factors, and telomere length for the entire sample, and individually by race.

For the first hypothesis, we hypothesized that CMS and/or CMS risk factors would be associated with telomere length, based on linear regression models run separately for black and white women. The presence of CMS and CMS risk factors was tested in models run separately for black and white women. We examined cross-sectional associations with CMS and CMS risk factor presence as predictors of telomere length. Additionally, we assessed prospective associations, examining how CMS and CMS risk factor presence at baseline and the follow-up for the period from 1996 to 1997 predicted telomere length at baseline and follow-up for the period from 1997 to 1998.

The second hypothesis posed that PA participation would be associated with telomere length differences in black and white women. To test the hypothesis, linear regression models were run separately for black women and white women. Women were grouped by PA participation (moderate-intensity PA <150 minutes per week/≥150 minutes per week and vigorous-intensity PA <75 minutes per week/≥75 minutes per week). Telomere length was then assessed for each group baseline and the 1997 to 1998 follow-up visit.

We further examined how CMS and/or CMS risk factor prevalence may impact the association between PA and telomere length, exploring whether difference in CMS risk factor prevalence in black and white women could explain differences in this relationship. To test this, women were categorized based on whether they met moderate-intensity PA or vigorous-intensity PA guidelines. Cross-sectional and prospective associations were examined using telomere length measurements at both baseline and the 1997 to 1998 follow-up visit. CMS and CMS risk factors were measured at baseline and follow-up in 1996 to 1997, and were individually added to each model as potential influences to better understand their role in the association between PA and telomere length. Models were run separately for black and white women. Analyses were performed using IBM SPSS for Windows software (Version 29, IBM Corp., Armonk, NY, USA) and a .05 significance level used in all models.

## Results

### Descriptive Characteristics

Descriptive characteristics for the study sample are detailed in Table 1. The total number of women analyzed was 417, with an age range of 65.0 to 87.0 years (M = 73.8). Black women made up 14.1% of the sample, while white women comprised 85.9% of the total sample. Black women were younger (M = 71.3 years) compared to white women (M = 74.2 years; *t* = 4.63, *P* < .001, d = 0.66) and black women had higher BMI (M = 29.4 kg/m^2^) compared to white women (M = 26.6 kg/m^2^; *t* = −3.98, *P* < .001, d = 0.52). Within the sociodemographic characteristics, white women were significantly more likely to be married compared to black women, who more frequently reported being separated, divorced, or widowed (χ^2^ = 18.96, *P* < .001). There were no significant differences among education level (χ^2^ = 2.80, *P* = .42), household income (χ^2^ = 5.04, *P* = .08), smoking status (χ^2^ = 1.27, *P* = .53), and alcohol use (χ^2^ = 2.94, *P* = .09) between the two groups.

**Table 1.** Participant Characteristics and Bivariate Associations for Sample and By Race in the Cardiovascular Health Study.

Sociodemographic characteristics	All women (N = 417)	Black women (n = 59)	White women (n = 358)	χ^2^/*t*	*P*
Age, M (SD)	73.8 (4.4)	71.3 (4.5)	74.2 (4.3)	4.63	<.001
Age range	65-87	65-83	68-87		
BMI, M (SD), kg/m^2^	27.0 (5.0)	29.4 (5.9)	26.6 (4.7)	−3.98	<.001
BMI range	15.8-46.3	15.8-46.3	16.7-44.6		
Education				2.80	.42
<HS, %	21.6	28.8	20.4		
HS graduate, %	31.9	27.1	32.7		
Some college, %	24.7	20.3	25.4		
>College, %	21.8	23.7	21.5		
Marital status				18.96	<.001
Single, %	6.5	5.1	6.7		
Married, %	57.1	37.3	60.3		
Separated/divorced, %	4.1	10.1	4.2		
Widowed, %	32.4	47.5	29.9		
Household income				5.04	0.08
<$5000-$24 999, %	60.1	72.7	58.0		
$25 000-$49 999, %	27.3	21.8	28.2		
≥$50 000, %	12.6	5.5	13.8		
Smoking status				1.27	0.53
Never, %	54.0	50.8	54.5		
Former, %	33.6	32.3	33.8		
Current, %	12.5	16.9	11.7		
Alcohol use, drink/day				2.94	0.09
<1 Alcohol drink, %	93.0	98.3	92.2		
≥1 Alcohol drink, %	7.0	1.7	7.8		

Valid percents are reported.

Abbreviations: BMI, body mass index; HS, high school.

In the total sample, 53.2% of participants met the recommendations for moderate-intensity PA as opposed to 46.8% that did not meet them, while the majority of women did not meet the recommendations for vigorous-intensity PA per week (95.0% vs 5.0%). There were no significant associations between moderate-intensity PA (χ^2^ = 0.92, *P* = .34) or vigorous-intensity PA adherence (χ^2^ = 1.60, *P* = .21) when examining by race. When examining bivariate associations for CMS risk factor prevalence, black women were significantly more likely to have high diastolic blood pressure (χ^2^ = 4.41, *P* = .04) and high fasting blood glucose (χ^2^ = 5.94, *P* = .02) at baseline visits compared to white women, but significantly less likely to have high triglycerides (χ^2^ = 8.21, *P* = .004) at baseline compared to white women. Similarly, at follow-up visits for the period from 1996 to 1997, black women were significantly more likely to have high diastolic blood pressure (χ^2^ = 4.38, *P* = .04) and high fasting blood glucose (χ^2^ = 4.23, *P* = .04) than white women. The mean telomere length for the sample was 6.38 kbp (±0.58), with black women having longer telomere length than white women at both baseline (6.59 kbp vs 6.34 kbp; *t*[415] = −3.15; *P* = .002, d = 0.42) and follow-up for the period from 1997 to 1998 (6.47 kbp vs 6.20 kbp; *t*[415] = −3.33; *P* < .001, d = 0.46). Descriptive characteristics and bivariate analyses for the study variables can be found in Table 2.

**Table 2.** Study Variables and Bivariate Associations for Sample and By Race in the Cardiovascular Health Study.

Variable	All women (n = 417)	Black women (n = 59)	White women (n = 358)	χ^2^/*t*	*P*
Physical activity measure, 1992-1993					
MPA, min/week, M (SD)	221.7 (216.9)	156.7 (190.2)	226.9 (218.0)	0.92	.34
MPA range	0-1240	0-1240	0-1065		
<150 min/week, %	46.8	52.5	45.8		
≥150 min/week, %	53.2	47.5	54.2		
VPA, min/week, M (SD)	8.4 (31.3)	3.9 (15.2)	9.2 (33.2)	1.60	.21
VPA range	0-240	0-90	0-240		
<75 min/week, %	95.0	98.3	94.4		
≥75 min/week, %	5.0	1.7	5.6		
Cardiometabolic syndrome risk factor measures, 1992-1993					
WC, in, M (SD)	37.9 (5.5)	39.2 (6.0)	37.7 (5.3)		
Increased WC				0.82	.37
Absent, %	30.5	25.4	31.3		
Present, %	69.5	74.6	68.7		
SBP, mmHg, M (SD)	135.4 (20.5)	138.3 (20.7)	135.0 (20.4)		
Elevated SBP				0.01	.94
Absent, %	24.5	24.1	24.6		
Present, %	75.5	75.9	75.4		
DBP, mmHg, M (SD)	70.3 (12.2)	75.9 (10.6)	69.4 (12.3)		
Elevated DBP				4.41	.04
Absent, %	43.7	31.0	45.8		
Present, %	56.3	69.0	54.2		
Elevated BP				0.44	.51
Absent, %	24.2	20.7	24.7		
Present, %	75.8	79.3	75.3		
FBG, mg/dL, M (SD)	102.7 (21.1)	109.8 (31.2)	101.6 (19.0)		
Increased FBG				5.94	.02
Absent, %	56.9	42.1	59.3		
Present, %	43.1	57.9	40.7		
TG, mg/dL, M (SD)	145.4 (63.8)	122.8 (56.9)	149.2 (64.2)		
Increased TG				8.21	.004
Absent, %	55.0	72.4	52.2		
Present, %	45.0	27.6	47.8		
HDL, mg/dL, M (SD)	56.6 (13.6)	60.2 (14.9)	56.0 (13.3)		
Decreased HDL				1.70	.19
Absent, %	69.0	76.3	67.8		
Present, %	31.0	23.7	32.2		
CMS, M (SD)	2.6 (1.4)	2.6 (1.4)	2.6 (1.4)	0.003	.96
Absent, %	47.1	47.4	47.0		
Present, %	52.9	52.6	53.0		
Cardiometabolic syndrome risk factor measures, 1996-1997					
WC, in, M (SD)	37.7 (5.5)	39.3 (5.7)	37.4 (5.4)		
Increased WC				1.51	.22
Absent, %	30.7	23.6	31.9		
Present, %	69.3	76.4	68.1		
SBP, mmHg, M (SD)	137.1 (20.4)	135.2 (17.4)	137.5 (20.9)		
Elevated SBP				0.90	.34
Absent, %	22.4	17.5	23.2		
Present, %	77.6	82.5	76.8		
DBP, mmHg, M (SD)	69.1 (10.9)	70.1 (11.7)	68.6 (10.7)		
Elevated DBP				4.38	.04
Absent, %	46.1	33.3	48.3		
Present, %	53.9	66.7	51.7		
Elevated BP					
Absent, %	18.9	12.1	20.1	2.07	.15
Present, %	81.1	87.9	79.9		
FBG, mg/dL, M (SD)	101.3 (21.4)	108.2 (28.8)	100.3 (19.9)		
Increased FBG				4.23	.04
Absent, %	62.4	50.0	64.4		
Present, %	37.6	50.0	35.6		
Telomere length measures					
TL, 1992-1993, kbp, M (SD)	6.38 (0.58)	6.59 (0.65)	6.34 (0.56)	−3.15	.002
TL range	4.76-8.67	5.39-8.67	4.76-8.20		
TL, 1997-1998, kbp, M (SD)	6.24 (0.59)	6.47 (0.62)	6.20 (0.57)	−3.33	<.001
TL range	4.60-8.49	5.16-8.49	4.60-8.17		

Valid percents are reported.

Abbreviations: BP, blood pressure; DBP, diastolic blood pressure; FBG, fasting blood glucose; HDL, high-density lipoprotein; MPA, moderate-intensity physical activity; PA, physical activity; SBP, systolic blood pressure; TG, triglycerides; TL, telomere length; VPA, vigorous-intensity physical activity; WC, waist circumference.

### Linear Regression Models

Table 3 summarizes the findings for hypothesis 1, whose anticipated associations would be present between CMS and CMS risk factors and telomere length among black and white women. The linear regression models focused on cross-sectional associations between CMS risk factors and telomere length at baseline, as well as prospective associations between CMS risk factors at baseline and telomere length at the 1997 to 1998 follow-up visit. At baseline, there was a significant association found in black women between the high-density lipoprotein <50 mg/dL and longer telomere length (*P* = .04), which remained significant even after adjusting for age (*P* = .02). An association was also present in black women with low high-density lipoprotein (<50 mg/dL) at baseline to longer telomere length at the 1997 to 1998 follow-up visit (*B* = 0.41, SE = 0.19, *P* = .02), which also remained significant after adjusting for age (*B* = 0.46, SE = 0.19, *P* = .01). In white women, a significant association was initially observed between high waist circumference (>35 inches) at baseline visit and increased telomere length at the 1997 to 1998 follow-up visit (*B* = 0.14, SE = 0.07, *P* = .03), but the association lost significance after adjusting for age (*B* = 0.12, SE = 0.06, *P* > .05). No other significant associations were identified for either black or white women (*p* > .05). A statistical interaction was calculated which demonstrated a statistically significant interaction was present between high-density lipoprotein and telomere length at baseline (*P* = .01) and follow-up for the period from 1997 1998 (*P* = .01) comparing black and white women. No significant statistical interaction effect was present among waist circumference, blood pressure, fasting blood glucose, triglycerides, and CMS when comparing by race.

**Table 3.** Linear Regression for Cardiometabolic Syndrome, Risk Factors, and Telomere Length.

Variable	Black women		White women	
	Unadjusted	Adjusted	Unadjusted	Adjusted
	B (SE)	B (SE)	B (SE)	B (SE)
Telomere length, 1992-1993				
Cardiometabolic syndrome and risk factors, 1992-1993				
Increased WC	0.15 (0.20)	0.03 (0.20)	0.12 (0.06)	0.11 (0.07)
Elevated BP	−0.05 (0.21)	−0.04 (0.21)	0.03 (0.07)	0.07 (0.07)
Increased FBG	0.10 (0.18)	0.03 (0.18)	−0.10 (0.06)	−0.09 (0.06)
Increased TG	−0.07 (0.19)	−0.06 (0.19)	−0.02 (0.06)	−0.04 (0.06)
Decreased HDL	**0.41 (0.19)**	**0.46 (0.19)**	−0.07 (0.06)	−0.09 (0.06)
CMS	−0.01 (0.18)	−0.002 (0.18)	0.03 (0.06)	0.02 (0.06)
Telomere length, 1997-1998				
Cardiometabolic syndrome and risk factors, 1992-1993				
Increased WC	0.16 (0.19)	0.08 (0.20)	**0.14 (0.07)**	0.12 (0.06)
Elevated BP	0.02 (0.20)	0.02 (0.20)	−0.05 (0.07)	0.002 (0.07)
Increased FBG	0.11 (0.17)	0.06 (0.17)	−0.05 (0.06)	−0.04 (0.06)
Increased TG	−0.09 (0.18)	−0.08 (0.18)	0.03 (0.06)	0.01 (0.06)
Decreased HDL	**0.46 (0.18)**	**0.50 (0.18)**	−0.04 (0.07)	−0.06 (0.06)
CMS	0.03 (0.17)	0.04 (0.17)	0.06 (0.06)	0.04 (0.06)
Cardiometabolic syndrome risk factors, 1996-1997				
Increased WC	0.38 (0.20)	0.35 (0.20)	0.09 (0.07)	0.07 (0.07)
Elevated BP	−0.07 (0.25)	−0.01 (0.25)	0.02 (0.08)	0.09 (0.08)
Increased FBG	0.22 (0.16)	0.20 (0.16)	−0.01 (0.07)	−0.02 (0.06)

Bold face type indicates a significant relationship between the variables. Adjusted for age.

Abbreviations: BP, blood pressure; FBG, fasting blood glucose; HDL, high-density lipoprotein; TG, triglycerides; WC, waist circumference.

Hypothesis 2 posited that PA participation would be associated with telomere length variations in black and white women. Analyses from the linear regression models, examining both cross-sectional and prospective associations between PA participation at baseline to telomere length at both baseline and the 1997 to 1998 follow-up visit, demonstrated no significant associations between either moderate-intensity PA or vigorous-intensity PA and telomere length in either black or white women at any time point (*P* > .05). A statistical interaction effect was calculated to examine the impact of PA on telomere length based on race and found no statistical significance for either moderate-intensity PA or vigorous-intensity PA (*P* > .05). Results are detailed in Table 4. Further, it was expected to reveal race-specific differences in PA participation and telomere length at baseline and follow-up for the period from 1997 to 1998 that were associated with differences in CMS risk factor prevalence. The analysis did not find a significant association in black or white women between PA and telomere length, when accounting for CMS risk factors (*P* > .05). Lastly, a statistical interaction was run to determine the significance of associations between PA participation and telomere length when accounting for CMS risk factor prevalence, and no statistically significant interaction effect was present between any CMS risk factor when comparing black and white women (*P* > .05). Results from this analysis can be found in Table S1.

**Table 4.** Linear Regression for Physical Activity and Telomere Length Accounting for Cardiometabolic Risk Factors.

Variable	Black women				White women			
	MPA		VPA		MPA		VPA	
	Unadjusted	Adjusted	Unadjusted	Adjusted	Unadjusted	Adjusted	Unadjusted	Adjusted
	B (SE)	B (SE)	B (SE)	B (SE)	B (SE)	B (SE)	B (SE)	B (SE)
Telomere length, 1992-1993								
Cardiometabolic syndrome risk factors, 1992-1993								
Increased WC	0.07 (0.17)	0.05 (0.17)	0.77 (0.67)	0.72 (0.65)	0.03 (0.06)	0.01 (0.06)	0.08 (0.13)	0.04 (0.13)
Elevated BP	0.09 (0.18)	0.07 (0.18)	0.83 (0.69)	0.74 (0.67)	0.02 (0.06)	0.01 (0.06)	0.09 (0.13)	0.05 (0.13)
Increased FBG	0.07 (0.18)	0.06 (0.17)	0.76 (0.68)	0.70 (0.66)	0.02 (0.06)	0.01 (0.06)	0.10 (0.13)	0.05 (0.13)
Elevated TG	0.08 (0.18)	0.06 (0.17)	0.78 (0.68)	0.70 (0.66)	0.02 (0.06)	0.01 (0.06)	0.09 (0.13)	0.05 (0.13)
Decreased HDL	0.08 (0.17)	0.06 (0.16)	0.90 (0.64)	0.82 (0.61)	0.02 (0.06)	0.004 (0.06)	0.09 (0.13)	0.05 (0.13)
CMS	0.08 (0.18)	0.08 (0.17)	0.92 (0.73)	1.02 (0.69)	0.03 (0.06)	0.01 (0.06)	0.08 (0.13)	0.04 (0.13)
Telomere length, 1997-1998								
Cardiometabolic syndrome risk factors, 1992-1993								
Increased WC	0.02 (0.16)	0.003 (0.16)	0.76 (0.64)	0.72 (0.63)	0.03 (0.06)	0.01 (0.06)	0.16 (0.13)	0.01 (0.13)
Elevated BP	0.02 (0.17)	0.01 (0.17)	0.88 (0.66)	0.81 (0.65)	0.02 (0.06)	−0.001 (0.06)	0.17 (0.13)	0.12 (0.13)
Increased FBG	−0.004 (0.17)	−01 (0.17)	0.77 (0.65)	0.73 (0.64)	0.02 (0.06)	0.002 (0.06)	0.18 (0.13)	0.12 (0.13)
Elevated TG	0.001 (0.17)	−0.01 (0.17)	0.79 (0.64)	0.73 (0.64)	0.02 (0.06)	−0.002 (0.06)	0.18 (0.13)	0.12 (0.13)
Decreased HDL	0.03 (0.16)	0.02 (0.15)	0.91 (0.60)	0.84 (0.59)	0.02 (0.06)	−0.002 (0.06)	0.17 (0.13)	0.12 (0.13)
CMS	0.02 (0.17)	0.02 (0.16)	1.00 (0.68)	1.08 (0.65)	0.04 (0.06)	0.01 (0.06)	0.16 (0.13)	0.11 (0.13)
Cardiometabolic syndrome risk factors, 1996-1997								
Increased WC	−0.09 (0.18)	−0.10 (0.18)	0.77 (0.64)	0.72 (0.64)	0.02 (0.06)	0.002 (0.06)	0.18 (0.14)	0.12 (0.14)
Elevated BP	0.02 (0.17)	0.01 (0.17)	0.85 (0.69)	0.83 (0.68)	0.02 (0.06)	−0.002 (0.06)	0.19 (0.14)	0.12 (0.14)
Increased FBG	−0.05 (0.16)	−0.05 (0.16)	0.76 (0.62)	0.72 (0.63)	0.01 (0.06)	−0.002 (0.06)	0.18 (0.14)	0.13 (0.14)

No significant relationships were observed between the variables. Adjusted for age.

Abbreviations: BP, blood pressure; CMS, cardiometabolic syndrome; FBG, fasting blood glucose; HDL, high-density lipoprotein; MPA, moderate-intensity physical activity; TG, triglycerides; VPA, vigorous-intensity physical activity; WC, waist circumference.

## Discussion

The goal of this investigation was to explore the associations of PA and CMS and CMS risk factors with telomere length, among black and white women. The study was guided by Kang’s Expanded Biobehavioral Model, which conceptualizes health outcomes as shaped by interactions across individual, behavioral, and biological domains. This framework informed the selection of variables and the decision to stratify analyses by race, providing a structure for examining how lifestyle factors like PA and clinical indicators of metabolic risk relate to cellular aging. The analysis did not find a significant association between moderate-intensity PA or vigorous-intensity PA and telomere length in either black or white women. However, there was a significant association in black women between high-density lipoprotein <50 mg/dL and longer telomere length both cross-sectionally and prospectively.

This study was designed to examine associations between physical activity and cardiometabolic risk factors, as well as telomere length, with models stratified by race. While only a limited number of significant associations emerged, the analyses were exploratory in nature and intended to generate hypotheses for future research. Findings should therefore be interpreted cautiously and in the context of this broader goal.

The significant associations in black women between high-density lipoprotein <50 mg/dL and longer telomere length were somewhat unexpected. Higher high-density lipoprotein levels have been reported among black individuals compared to white populations,^55^ and prior research has suggested high-density lipoprotein has a protective effect on telomere length,^19,56^ which would suggest the inverse relationship to be suspected. However, there is evidence high-density lipoprotein levels may decline after menopause secondary to the drop in estrogen levels.^57,58^ The findings may demonstrate a decrease in high-density lipoprotein related to the decline in estrogen for postmenopausal women. Though no similar findings were found in white women, black women had significantly longer telomere length at baseline and the 1997 to 1998 follow-up visit compared to white women, which may account for this variation. It is also possible the association between low high-density lipoprotein and increased telomere length may be related to confounders not accounted for in this analysis. Lastly, it is feasible the finding is a spurious association that demonstrates a sampling bias in black women who participated in research at that time or is secondary to selective survival, suggesting the black women who participated in the study may be unique and not representative of the population at the time of data collection. Future research should further investigate the association between low high-density lipoprotein and longer telomere length, using larger cohorts of older black women.

The lack of significant associations between moderate-intensity PA or vigorous-intensity PA and telomere length in both racial groups was surprising and challenged the initial expectations that increased PA would be associated with longer telomere length. Moreover, the relationship remained statistically non-significant even after accounting for CMS and/or CMS risk factors. Although some studies^59,60^ reported a similar lack of significant associations, others have found significant links, particularly in older women.^44,45^ However, these latter studies examined moderate-to-vigorous physical activity as a combined measure. Research specifically focusing on moderate-intensity PA is limited, though some evidence suggests that moderate-intensity PA may help preserve telomere length in women.^61^ On the other hand, several studies have consistently supported the notion that more vigorous activities are associated with longer telomere length,^62,63^ though only one reported findings specific to women.^47^

It is possible there may be a discrepancy between the self-reported PA in both duration and intensity and the actual time and energy expended by the participants. Moreover, the low validity of the PA measure may further impact the reliability of the findings. Physical activity levels tend to be low among older adults, which may help contextualize the largely null findings. Although the self-report measure used has demonstrated validity in older populations, it may still underestimate or inaccurately capture physical activity, especially at higher intensities. Moreover, the study did not include data on sedentary behavior which limits the ability to fully assess the relationship between activity patterns and telomere length.

It may also be that the recommended intensity and duration of PA per week (≥150 minutes of moderate-intensity PA or ≥75 minutes of vigorous-intensity PA), may not be sufficient for eliciting positive telomere length change in older black and white women. Lastly, it is feasible that confounders not accounted for may be responsible for the lack of significant associations observed in the study. Future research should include larger cohorts of older black and white women that examine the effects of objectively-measured PA on telomere length to further elucidate the relationship between PA and telomere length in older women.

The study found that older black women had longer telomere length than older white women, despite having significantly higher rates of high diastolic blood pressure and high fasting blood glucose. These CMS risk factors have previously been associated with shorter telomere length.^64-66^ The longer telomere length findings in black women identified corroborate similar research findings suggesting that despite age-related attrition, black women maintain longer telomere length than white women.^20,21^ The reasons for the telomere length disparity remain unclear, with some proposing that genetic variations may explain the racial differences in telomere length,^67,68^ while others suggest that social contexts and disadvantages may influence telomere length across races.^69^

These findings should be considered through the lens of Kang’s Expanded Biobehavioral Model, which illustrates the complex interactions among individual (age, telomere length), behavioral (PA), and biological (waist circumference, blood pressure, fasting blood glucose, triglycerides, high-density lipoproteins) domains in shaping health outcomes (CMS or CMS risk factor prevalence). This framework informed the study’s race-stratified design and variable selection, providing a structure for interpreting how differences in behavioral and biological factors may contribute to aging disparities. Although education, marital status, and income were not significantly associated with key outcomes in this study, the observed relationships among PA, CMS, and telomere length support the utility of the model for examining these interrelated factors. However, the interpretation of racial differences in telomere length is limited by the absence of key contextual variables. Stressors such as racism and discrimination are known to influence biological aging, including telomere length, but data on these exposures were not available in this data set. Their absence limits how well we can understand the factors contributing to telomere differences between racial groups. This represents a meaningful gap and underscores the need for future studies that directly include these variables. Future research should incorporate additional domains from the model, including social stress, socioeconomic status, and diet, to further elucidate the complex pathways linking behavior and biology to cellular aging in diverse populations.

### Strengths and Limitations

Examination of PA, CMS risk factors, and telomere length in women within the CHS has not previously been conducted, rendering the presented research the first of its kind. PA data were collected using a questionnaire that has demonstrated reliability in older adults.^70^ Anthropometric measures and blood samples were collected by trained research staff through standardized objective measurement techniques, which improves the consistency and validity of the measures and accuracy of the findings. Use of medications for three of the risk factors (hypertension, type II diabetes, dyslipidemia) was included in the analysis for CMS risk factors, appropriately accounting for CMS risk factor prevalence. The data set provided CMS risk factors and telomere length measures from two time points, allowing for both cross-sectional and longitudinal analysis.

Given the secondary analysis nature of the study, analysis was limited to the data available. Use of an existing data set affected the ability to analyze all 5 CMS risk factors at the 1996 to 1997 follow-up visit as only 3 were collected, as well as additional confounders that may impact telomere length. Additionally, we were unable to adjust for certain potential confounders that may influence telomere length, such as cognitive status or the presence of other chronic conditions. These factors may contribute to residual confounding and should be considered in future research using more comprehensive data. The PA data was limited to self-report, which may be less reliable than objective PA measures^71^ and has low content validity for populations of women.^50^ Moreover, the data set did not include information on sedentary behavior, which could influence associations between physical activity and cellular aging. This represents an additional unmeasured factor that should be considered in future research.

At the time of participant enrollment in the CHS (late 1980s to early 1990), race was primarily categorized as black or white, and only women from these 2 groups were included in this analysis. Participants from other racial or ethnic backgrounds were not retained due to small sample size (<1% of the sample), which constrained the ability to draw meaningful comparisons. The small number of black women included in the study was a limitation in the third analysis, when examining the association between PA and telomere length, accounting for all CMS risk factors. The noted limitation weakened the statistical power of the findings, suggesting that more emphasis should be placed on the results from the models that investigated the individual CMS risk factors rather than the collective analysis. In addition, given the number of models tested across CMS risk factors, PA types, timepoints, and race groups, the potential for type I error should be considered. Although the number of comparisons reflects the study’s primary aims, the limited number of significant findings highlights the need for cautious interpretation and replication in future studies. Further, since the study focused exclusively on older black and white women, the findings cannot be reliably extrapolated to other age groups, races, or to men. Caution should be used with interpretation of the results.

## Conclusion

There was no evidence of a significant relationship between PA and telomere length in black or white women demonstrated in the research findings, nor was the lack of association impacted when accounting for all CMS risk factors. The lack of findings challenges the initial hypotheses that PA universally affects telomere length, suggesting a more complex interplay involving PA, cardiometabolic health, and telomere length. However, the analysis did demonstrate a significant association between low high-density lipoprotein levels and longer telomere length in black women, which may be associated with an age-related decline in high-density lipoprotein specific to post-menopausal black women with significantly longer telomere length than their white counterparts, unaccounted confounders, or a spurious association of the population resulting in a sampling bias. Additional research is needed to explore the effects of PA on telomere length for both older black and white women and to further investigate the implications of low high-density lipoprotein on telomere length in older black women.

## Supplemental Material

sj-pdf-1-wjn-10.1177_01939459251387798 – Supplemental material for Physical Activity, Telomere Length, and Cardiometabolic Syndrome in Older Women: The Cardiovascular Health Study Supplemental material, sj-pdf-1-wjn-10.1177_01939459251387798 for Physical Activity, Telomere Length, and Cardiometabolic Syndrome in Older Women: The Cardiovascular Health Study by Jeni Page, Melissa Richard, Elizabeth Lyons, Elizabeth Baumler, M. Terese Verklan, Michelle C. Odden and Elizabeth Lorenzo in Western Journal of Nursing Research
