# Supplementary material for: Physical Activity, Telomere Length, and Cardiometabolic Syndrome in Older Women: The Cardiovascular Health Study
Source: West J Nurs Res. 2025 Nov 29;48(1):77–89. doi: 10.1177/01939459251387798 (PMC12728086; doi:10.1177/01939459251387798)
Supplement: sj-pdf-1-wjn-10.1177_01939459251387798 – Supplemental material for Physical Activity, Telomere Length, and Cardiometabolic Syndrome in Older Women: The Cardiovascular Health Study [file sj-pdf-1-wjn-10.1177_01939459251387798.pdf]

Supplemental Table S1

Linear Regression for Physical Activity and Telomere Length Accounting for Cardiometabolic Risk Factors

	Black women				White women			
	MPA		VPA		MPA		VPA	
	Unadjusted B (SE)	Adjusted B (SE)	Unadjusted B (SE)	Adjusted B (SE)	Unadjusted B (SE)	Adjusted B (SE)	Unadjusted B (SE)	Adjusted B (SE)
Telomere length, 1992-93								
Cardiometabolic syndrome risk factors, 1992-93								
Increased WC	0.07 (0.17)	0.05 (0.17)	0.77 (0.67)	0.72 (0.65)	0.03 (0.06)	0.01 (0.06)	0.08 (0.13)	0.04 (0.13)
Elevated BP	0.09 (0.18)	0.07 (0.18)	0.83 (0.69)	0.74 (0.67)	0.02 (0.06)	0.01 (0.06)	0.09 (0.13)	0.05 (0.13)
Increased FBG	0.07 (0.18)	0.06 (0.17)	0.76 (0.68)	0.70 (0.66)	0.02 (0.06)	0.01 (0.06)	0.10 (0.13)	0.05 (0.13)
Elevated TG	0.08 (0.18)	0.06 (0.17)	0.78 (0.68)	0.70 (0.66)	0.02 (0.06)	0.01 (0.06)	0.09 (0.13)	0.05 (0.13)
Decreased HDL	0.08 (0.17)	0.06 (0.16)	0.90 (0.64)	0.82 (0.61)	0.02 (0.06)	0.004 (0.06)	0.09 (0.13)	0.05 (0.13)
CMS	0.08 (0.18)	0.08 (0.17)	0.92 (0.73)	1.02 (0.69)	0.03 (0.06)	0.01 (0.06)	0.08 (0.13)	0.04 (0.13)
Telomere length, 1997-98								
Cardiometabolic syndrome risk factors, 1992-93								
Increased WC	0.02 (0.16)	0.003 (0.16)	0.76 (0.64)	0.72 (0.63)	0.03 (0.06)	0.01 (0.06)	0.16 (0.13)	0.01 (0.13)
Elevated BP	0.02 (0.17)	0.01 (0.17)	0.88 (0.66)	0.81 (0.65)	0.02 (0.06)	-0.001 (0.06)	0.17 (0.13)	0.12 (0.13)
Increased FBG	-0.004 (0.17)	-01 (0.17)	0.77 (0.65)	0.73 (0.64)	0.02 (0.06)	0.002 (0.06)	0.18 (0.13)	0.12 (0.13)
Elevated TG	0.001 (0.17)	-0.01 (0.17)	0.79 (0.64)	0.73 (0.64)	0.02 (0.06)	-0.002 (0.06)	0.18 (0.13)	0.12 (0.13)
Decreased HDL	0.03 (0.16)	0.02(0.15)	0.91 (0.60)	0.84 (0.59)	0.02 (0.06)	-0.002 (0.06)	0.17 (0.13)	0.12 (0.13)
CMS	0.02 (0.17)	0.02 (0.16)	1.00 (0.68)	1.08 (0.65)	0.04 (0.06)	0.01 (0.06)	0.16 (0.13)	0.11 (0.13)
Cardiometabolic syndrome risk factors, 1996-97								
Increased WC	-0.09 (0.18)	-0.10 (0.18)	0.77 (0.64)	0.72 (0.64)	0.02 (0.06)	0.002 (0.06)	0.18 (0.14)	0.12 (0.14)
Elevated BP	0.02 (0.17)	0.01 (0.17)	0.85 (0.69)	0.83 (0.68)	0.02 (0.06)	-0.002 (0.06)	0.19 (0.14)	0.12 (0.14)
Increased FBG	-0.05 (0.16)	-0.05 (0.16)	0.76 (0.62)	0.72 (0.63)	0.01 (0.06)	-0.002 (0.06)	0.18 (0.14)	0.13 (0.14)

Note. MPA = moderate intensity physical activity; VPA = vigorous intensity physical activity; WC = waist circumference; BP = blood pressure; FBG = fasting blood glucose; TG = triglycerides; HDL = high-density lipoprotein; CMS = cardiometabolic syndrome. Bold face type indicates a significant relationship between the variables. Adjusted for age.
